# Development and optimisation of a preclinical cone beam computed tomography-based radiomics workflow for radiation oncology research

**DOI:** 10.1016/j.phro.2023.100446

**Published:** 2023-05-16

**Authors:** Kathryn H. Brown, Neree Payan, Sarah Osman, Mihaela Ghita, Gerard M. Walls, Ileana Silvestre Patallo, Giuseppe Schettino, Kevin M. Prise, Conor K. McGarry, Karl T. Butterworth

**Affiliations:** a Patrick G. Johnston Centre for Cancer Research, Queen’s University Belfast, Northern Ireland, UK; b University College London Hospitals NHS Foundation Trust Department of Radiotherapy, London, UK; c Cancer Centre, Belfast Health & Social Care Trust, Lisburn Road, Belfast BT9 7AB, Northern Ireland, UK; d National Physical Laboratory, London, UK

**Keywords:** Radiomics, CBCT imaging, Preclinical models, Standardisation, Workflow development

## Abstract

**Background and purpose:**

Radiomics features derived from medical images have the potential to act as imaging biomarkers to improve diagnosis and predict treatment response in oncology. However, the complex relationships between radiomics features and the biological characteristics of tumours are yet to be fully determined. In this study, we developed a preclinical cone beam computed tomography (CBCT) radiomics workflow with the aim to use *in vivo* models to further develop radiomics signatures.

**Materials and methods:**

CBCT scans of a mouse phantom were acquired using onboard imaging from a small animal radiotherapy research platform (SARRP, Xstrahl). The repeatability and reproducibility of radiomics outputs were compared across different imaging protocols, segmentation sizes, pre-processing parameters and materials. Robust features were identified and used to compare scans of two xenograft mouse tumour models (A549 and H460).

**Results:**

Changes to the radiomics workflow significantly impact feature robustness. Preclinical CBCT radiomics analysis is feasible with 119 stable features identified from scans imaged at 60 kV, 25 bin width and 0.26 mm slice thickness. Large variation in segmentation volumes reduced the number of reliable radiomics features for analysis. Standardization in imaging and analysis parameters is essential in preclinical radiomics analysis to improve accuracy of outputs, leading to more consistent and reproducible findings.

**Conclusions:**

We present the first optimised workflow for preclinical CBCT radiomics to identify imaging biomarkers. Preclinical radiomics has the potential to maximise the quantity of data captured in *in vivo* experiments and could provide key information supporting the wider application of radiomics.

## 1 Introduction

Medical imaging is central to clinical decision-making for the identification of tumours, delivery of treatment and follow-up assessments [1]. It is well established that these radiological images are data rich and can be used as imaging biomarkers [2]. With the commercialisation of parallel preclinical computed tomography (CT) and cone-beam CT (CBCT) imaging platforms onboard small animal irradiators [3]; imaging biomarkers can be determined from these preclinical scans [4], [5], [6].

Radiomics is a high-throughput form of image analysis to extract quantitative information from medical images which can be correlated to biological outcomes to improve diagnostic, prognostic and predictive accuracy [7], [8], [9], [10], [11]. Whilst radiomics has been termed a ‘virtual biopsy’ and associated with several clinical endpoints, the complex relationships between radiomics and clinical factors are still largely unknown [12]. Standardisation of image acquisition and analysis to identify and validate imaging biomarkers is a large focus within radiation oncology [13], [14], [15].

The prognostic potential of magnetic resonance (MR)- and CT-based clinical radiomics has already been well documented within the literature [1], [16], [17], [18], with emerging evidence of feasibility using CBCT scans [19], [20], [21], [22]. CBCT scans are acquired at multiple timepoints throughout radiotherapy treatment and extraction of radiomics signatures from these could lead to surplus data in both clinical and preclinical settings [23].

Previously, Panth *et al* demonstrated that mouse models can be used to expand our knowledge of CT-based radiomics signatures [24]. Since then, preclinical radiomics analysis has evolved to include CT, MRI and PET imaging for the detection and prediction of tumour phenotypes, early metastases and treatment response [24], [25], [26], [27]. However, preclinical radiomics lacks standardisation of methods and validation of results [28]. This is in addition to the lack of imaging standards and protocols which already exist within preclinical studies [29]. Repeatability and reproducibility analysis is therefore crucial to evaluate feature stability in a controlled scenario (test–retest) and the influence of different imaging acquisition or analysis parameters (scan-rescan) [30].

In this study, we assessed the repeatability and reproducibility of CBCT-based radiomics features toward standardising the first preclinical CBCT radiomics workflow. Different image acquisition protocols and feature extraction methods were trialled to identify a subset of features that are robust for analysis. These features were then applied to preclinical tumour models in a pilot feasibility analysis.

## 2 Materials and methods

### 2.1 Phantoms

Two phantoms were used in this study (Supplementary Fig. 1). Firstly, an anatomically correct, tissue-equivalent mouse phantom with densities and atomic composition for bone (1.39 g/cm^3^), lung (0.68 g/cm^3^) and soft tissue (1.01 g/cm^3^) was used for workflow analysis [31], [32]. Secondly, an in-house Perspex phantom (60x60x60 mm) with cylindrical inserts (20x60mm) for air, solid water (Bart’s) (1.05 g/cm^3^), PVC (1.47 g/cm^3^) and acetal (1.52 g/cm^3^) was used to compare how differences in material density effect texture features.

### 2.2 Imaging

CBCT imaging was performed using the Small Animal Radiation Research Platform (SARRP, Xstrahl Life Sciences, UK) (Supplementary Table 1). For the mouse phantom, scans were acquired twice at 40, 50 and 60 kV and 0.8 mA (0.5 mm Al filtration). For the texture phantom, scans were acquired twice at 60 kV. All energies had an imaging dose of 2.4 cGy.

### 2.3 Tumour models

CBCT scans from previous in vivo experiments were retrospectively analysed. Tumour xenograft studies were performed using the non-small cell lung cancer (NSCLC) cell lines, A549 and H460. Cells were cultured *in vitro* (Dulbecco’s modified Eagle’s medium (DMEM) supplemented with 10% foetal bovine serum and 1% penicillin/streptomycin) and prepared in phosphate-buffered saline (PBS) for subcutaneous injection into the flank of SCID mice. At 100 mm^3^, tumours were imaged at 60 kV on the SARRP (n = 9 for each arm). All experimental procedures were carried out in accordance with the Home Office Guidance on the Operation of the Animals (Scientific Procedures Act 1986) (PPL2813).

### 2.4 Segmentation

Segmentations were created using ITK-SNAP software (version 3.8.0) [33]. Manual contours were created using the 3-D round brush in the abdominal region of the mouse phantom model (not including lung or bone). Standard spherical segmentations of 27.68, 34.38, 41.71, 92.24 and 237.5 mm^3^ were used for scan-rescan analysis. Segmentation of tumours was completed using a standard spherical segmentation volume of 94.25 mm^3^. This method was adopted to reduce the impact of interobserver variabilities associated with manual contours [63].

### 2.5 Radiomics analysis

Radiomics analysis was performed using PyRadiomics (version 2.7.7, Harvard Medical School, Boston, MA, USA) [34], which is compliant with the Image Biomarker Standardisation Initiative (IBSI) [14]. 842 features were extracted including: shape (n = 14), first order statistics (n = 18), gray level cooccurrence matrix (GLCM) (n = 23), gray level run length matrix (GLRLM) (n = 16), gray level size zone matrix (GLSZM) (n = 16), gray level dependence matrix (GLDM) (n = 14) and neighbouring gray tone difference matrix (NGTDM) (n = 5). Wavelet filtering was also applied to these features. Shape features were only used for correlation analysis to segmentation volume.

To optimise our radiomics workflow, different pre-processing parameters were tested. The slice thickness of the CBCT scans were resampled to either 0.2, 0.26, 0.3, 0.5 or 1 mm by changing the “resampledPixelSpacing“, without modifying the axial spacing. Image intensity discretization was performed to compare different fixed bin width values of 10, 25, 50 and 100 by altering the “binWidth”.

### 2.6 Correlation to segmentation volume

Features highly correlated to volume changes was determined using correlation analysis (*cor* function within the *corrplot* library in RStudio software (version 4.1.2)). The Pearson correlation coefficient was calculated for each feature with respect to volume and a correlation coefficient > 0.8 applied.

### 2.7 Statistical analysis

The intraclass correlation coefficient (ICC) was used to determine the reliability and robustness of radiomics outputs through the production of a reliability index (Table 1). ICCs were calculated using the *irr* library from the *lpSolve* package in RStudio.

**Table 1:** Classification of ICC results. Koo *et al* classifies ICC as poor (<0.5), moderate (0.5–0.7), good (0.7–0.9) and excellent (>0.9) [35,36]. A stricter ICC of > 0.8 was used to determine good/excellent reliability to better match with previous thresholds reported in test–retest analysis. The 95% confidence intervals (CIs) (>0.7) were used to remove errors and indicate robustness as recommended by Koo *et al*.

Intraclass correlation coefficient (ICC)	Reliability Index
0.8	Good reliability
>0.8	Excellent reliability
1	Perfect reliability
Classification of ICC in this study	
ICC > 0.8 & 95% confidence interval > 0.7	Highly robust

Reliability analysis was based on a single value with absolute-agreement and determined using 2-way mixed-effects models for the scan-rescan analysis of radiomics feature outputs across each variable [35]. Reproducibility analysis was based on an average of each scan and rescan (n = 6) with absolute-agreement and determined using 2-way mixed-effects models. Analysis was conducted between the tumour cohorts’ through a 2-way mixed-effects ICC model. The Pearson correlation coefficient was also calculated for each feature (*cor* in RStudio) and a correlation coefficient > 0.8 was considered significant. Comparison of radiomics outputs for tumour models was performed using a paired *t*-test (two-tailed, *p* < 0.05) (n = 9). Analysis was performed using GraphPad Prism 7 (Version 7.0) with significance reported as *p* **** <0.0001.

## 3 Results

### 3.1 Repeatability of preclinical radiomics features

**Fig. 1:**
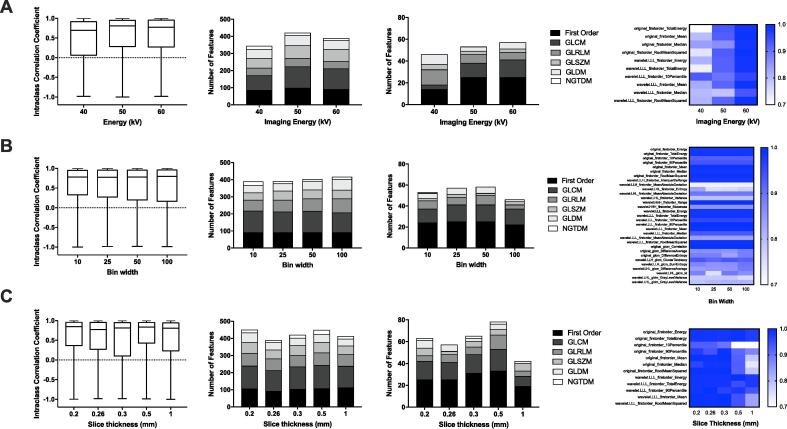
Reliability and robustness of radiomics features with varying CBCT image acquisition and image discretisation methods. CBCT scans of a 3-D mouse phantom were acquired on the SARRP and analysed using PyRadiomics. Boxplots display ICC values of radiomics features (left). The number of reliable radiomics features (ICC > 0.8) (middle-left). The number of robust radiomics features (lower CI > 0.7) (middle-right). Heatmap of ICC values for overlapping robust features (right). Panel A: Reliability of radiomics features across imaging energies of 40, 50 and 60 kV. Panel B: Reliability of radiomics features after changing the intensity discretization via bin width to 10, 25, 50 or 100. Panel C: Reliability of radiomics features after changing the slice thickness during analysis (0.2, 0.26, 0.3, 0.5 & 1 mm).

Repeatability was assessed using scan-rescans of a mouse phantom acquired at different imaging energies or processed using different bin widths or slice thickness. This analysis aims to show how differences in the preclinical radiomics workflow may reduce the reliability of features. For imaging energies of 40, 50 and 60 kV there were 343, 420 and 388 reliable features respectively (ICC > 0.8) (Fig. 1 A). However, only 46, 53 and 57 features were robust (lower CI of the ICC > 0.7). Scans acquired at 40 kV had the greatest variability; potentially due to increased artefacts and noise in scans. Only 10 robust features (1%) overlapped across all 3 imaging energies; all of which were first order features (Fig. 1 A). CBCT scans acquired at different imaging energies can therefore limit the number of robust radiomics features for comparative analysis and the higher energies (60 kV) recommended for analysis.

Repeatability of features across different bin widths was compared at 60 kV (Fig. 1 B). Bin widths of 25 and 50 had the most robust features of 57 and 58 (7%) respectively, 43 of which were shared. Fig. 1 B includes a heatmap of the 31 (4%) robust and reliable features maintained across all bin widths.

Radiomics features were extracted with a resampled slice thickness of 0.2, 0.26, 0.3, 0.5 or 1 mm (Fig. 1 C). A slice thickness of 0.5 mm had the most robust features of 78 (9%). Only 12 (1%) overlapping features were identified across all slice thicknesses, all of which were first order. Additional analysis showed that increasing the slice thickness led to variability in shape and volume analysis (“original_shape_MeshVolume”).

### 3.2 The volume effect

**Fig. 2:**
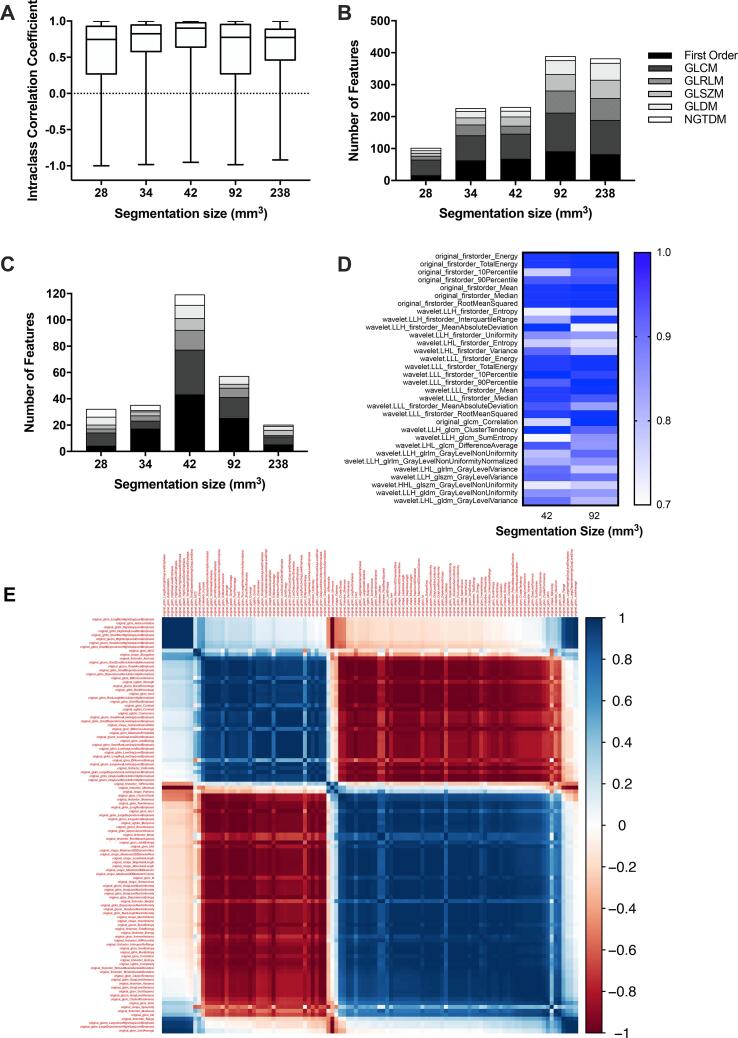
Overview of radiomics outputs for a range of segmentation volumes. Panel A: Boxplots to display ICC values of radiomics features assessed across a range of segmentation volumes (28 – 238 mm^3^). Panel B: The number of reliable radiomics features by feature class for each segmentation volume. Panel C: The number of robust features for each segmentation volume Panel D: Heatmap of overlapping robust features for 42 and 92 mm^3^ segmentation volumes. Panel E: Hierarchical correlation matrix to identify unfiltered radiomics features that are highly correlated to an increase in segmentation volume. 54 unfiltered features were highly correlated to changes in the segmentation volume.

To determine if volume impacts feature reliability or if there is a minimum volume suitable for extracting reliable results, we compared radiomics outputs for a range of volumes in a mouse phantom model. As preclinical models are smaller than their clinical counterparts five relevant volumes for preclinical analysis were used (28, 34, 42, 92 and 238 mm^3^) (Supplementary Fig. 2). The smallest volume, 28 mm^3^, had the least repeatable features (101 features), in comparison, larger volumes of 92 and 238 mm^3^ had 388 and 381 repeatable features respectively (Fig. 2 B). There was no overlap in robust features across the range of segmentation volumes evaluated. Supplementary Fig. 3 details overlapping features amongst similar volumes. These results suggest that volumes < 34 mm^3^ may be too small to extract reliable data.

The number of robust features did not increase with increasing segmentation volume. Volumes of 42 and 92 mm^3^ had the most robust and reliable features of 119 and 57 features respectively (Fig. 2 C). A volume range of 42–92 mm^3^ may be suitable for preclinical radiomics analysis with 32 features maintained for both volumes (Fig. 2 D). These non-linear results may be influenced by the phantom model used in which we assume tissue regions are homogeneous. Our results show that first order and GLDM features have a higher reliability range when comparing different volumes. Whereas GLCM, GLSZM and NGTDM features are more sensitive to volume changes (Supplementary Fig. 4).

The correlation of segmentation volume to unfiltered radiomics features is shown in Fig. 2 E. Fifty-four features were highly correlated to an increase in segmentation volume (original_shape_MeshVolume). These included 9 shape, 12 first order, 13 GLCM, 6 GLRLM, 6 GLSZM, 6 GLDM and 2 NGTDM features (Supplementary Table 2). Of these, 7 have been determined as reliable features from scan-rescan analysis for volumes of 42 – 92 mm^3^.

A workflow of scans imaged at 60 kV and features extracted at bin width of 25 and slice thickness maintained at 0.26 mm was determined. From repeatability analysis 119 (14%) robust features can be extracted at 42 mm^3^ (Supplementary Table 3) and 57 (7%) robust features at 92 mm^3^ (Supplementary Table 4) which are stable for preclinical analysis.

### 3.3 Reproducibility of preclinical radiomics features

**Fig. 3:**
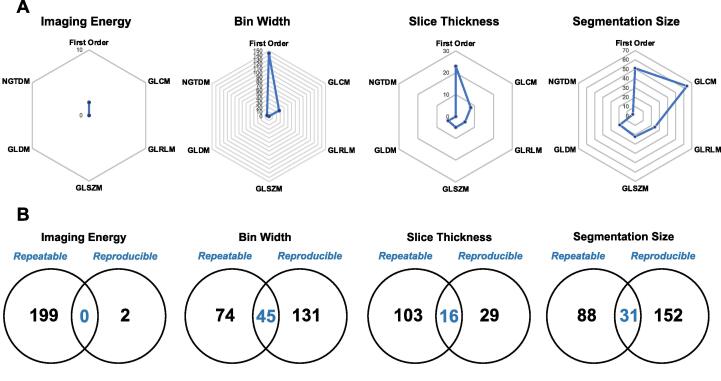
Results of the reproducibility analysis for preclinical radiomics. Panel A: Reproducibility of radiomics outputs was compared within each variable and the number of features with a good ICC (>0.8) was plotted for imaging energy, bin width, slice thickness and segmentation size. Panel B: Venn diagrams to show the overlap of repeatable and reproducible features extracted from a mouse phantom using varying preclinical radiomics analysis methods. Repeatable features include the 119 robust features detailed in Table 2.

To further optimise our results, we assessed the reproducibility of radiomics outputs. Changing the imaging energy had the biggest impact on the reproducibility of features with only 2 features identified. Altering the slice thickness resulted in 45 reproducible features. Variations in the bin width and segmentation sizes were least affected with 176 and 183 reproducible features respectively (Fig. 3 A). Overall, the most reproducible feature types were first order, GLCM and GLRLM.

No robust features overlapped from repeatability and reproducibility studies for varying imaging energies; however, there was an overlap of 45, 16 and 31 features for bin width, slice thickness and segmentation size respectively (Fig. 3 B). These features are therefore highly conserved for comparison of preclinical radiomics outputs when using different workflow parameters (Supplementary Table 5).

### 3.4 Texture analysis

**Fig. 4:**
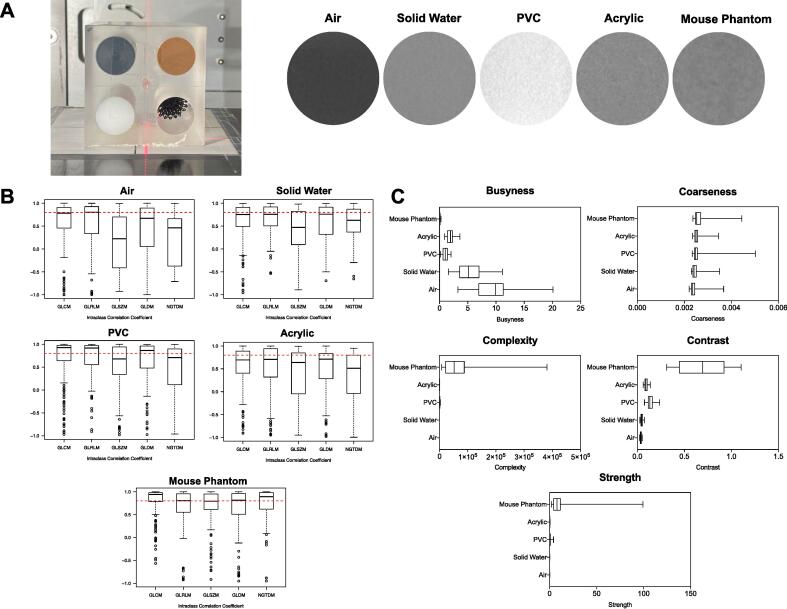
Radiomics features are affected by changes in texture. Panel A: Textural phantom on imaging bed with labels for each textural insert (left). CBCT cross section of the 4 different cylindrical inserts and the mouse phantom (right). The average gray level intensity (original_firstorder_Mean) for each material was 1861 for air, 2940 for solid water, 4138 for PVC, 2917 for acetal and 16,844 for the mouse phantom. Panel B: Boxplots of ICC outputs for wavelet radiomics features across textures at a segmentation volume of 42 mm^3^. Panel C: NGTDM feature values for air, solid water, PVC, acetal and the mouse phantom.

A multi-density phantom was used to measure the variability of radiomics features to changes in texture. Bart’s solid water (1.05 g/cm^3^) and the mouse phantom (1.01 g/cm^3^) have similar densities and visually look similar from CBCT scans yet the average gray level intensity (original_firstorder_Mean) values differ from 2,940 to 16,844 (Fig. 4 A). Scan-rescan analysis was conducted with ICC outputs for wavelet features shown in Fig. 4 B. GLSZM features had the lowest median ICC for all textures apart from acetal (Fig. 4 B). NGTDM features were further analysed and shown to be influenced by changes in density (Fig. 4 C). This confirms that preclinical radiomics analysis can be used to differentiate materials with differing density through textural radiomics analysis.

### 3.5 Differentiation of tumour models using radiomics features: Pilot analysis

**Fig. 5:**
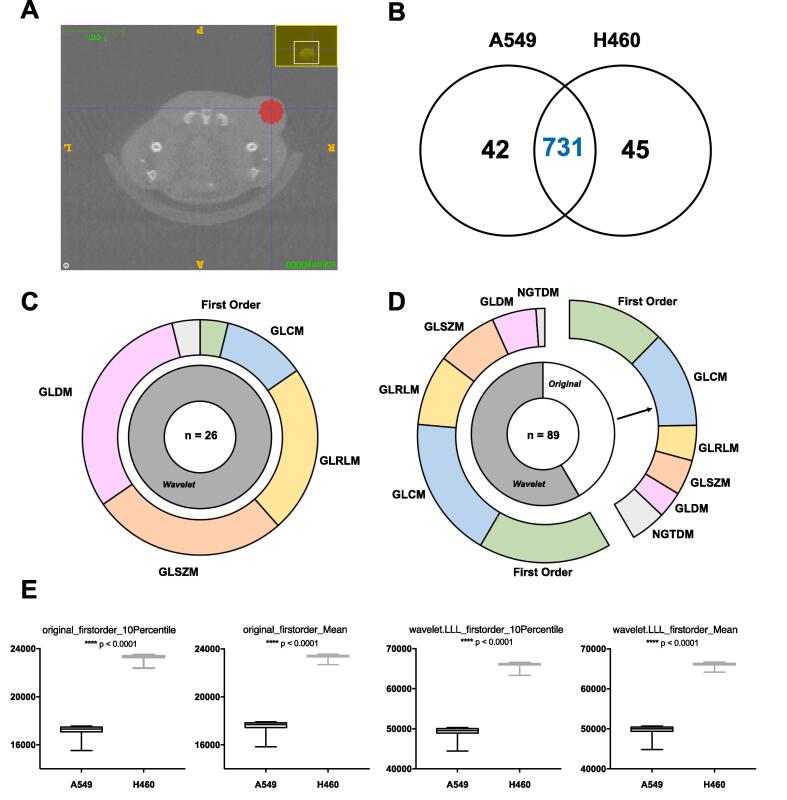
Application of radiomics analysis to preclinical CBCT scans of lung tumour models (A549 and H460). Panel A: Example of pre-treatment CBCT scan acquired at 60 kV used for analysis. An example of the spherical segmentation can be visualised in red. Panel B: Venn diagram of highly correlated features overlapping between tumour cohorts. Panel C: Schematic to represent the 26 reliable radiomics features for A549 tumours (ICC > 0.8) subdivided by feature type and class. Panel D: The 89 reliable radiomics features for H460 tumours (ICC > 0.8) broken down by feature type and class. Panel E: Example of 4 repeatable and reproducible radiomics features which can be used to differentiate the two tumour cohorts. Significance reported as *p* **** < 0.0001. (For interpretation of the references to colour in this figure legend, the reader is referred to the web version of this article.)

Pre-treatment CBCT scans from two NSCLC tumour models were retrospectively analysed (Fig. 5 A). There were 773 and 776 highly correlated features for A549 and H460 tumours respectively with 731 shared (Fig. 5B). Test-retest analysis identified 26 and 89 reliable features for the A549 and H460 cohorts respectively (Fig. 5 C/D). After comparison with robust features (Supplementary Table 5), 4 features can be used to differentiate A549 and H460 tumours on preclinical CBCT scans (Fig. 5 E).

## 4 Discussion

Since the first application of radiomics analysis for phenotype prediction, it has led to the discovery of imaging biomarkers and evolved to include multiple imaging modalities [1], [14], [23], [36], [37]. Radiomics analysis also has major clinical and economic benefits for the replacement of invasive and expensive procedures to determine tumour heterogeneity, such as biopsies [38]. Yet, real-world application of radiomics in oncology is limited by the lack of “big” and standardised clinical data due to different imaging protocols, variability in patient history and restrictions by law and ethics [39].

Mouse models are hugely beneficial in radiation oncology for the understanding of cancer progression and treatment development [40]. In addition, preclinical radiomics analysis has been successful using preclinical CT and MR scans [24], [25], [41]. Despite evidence that mouse models can expand our knowledge in radiomics signatures, there are currently no established guidelines to ensure consistency in preclinical analysis [28]. We aimed to optimise and standardise the first preclinical CBCT-radiomics workflow to improve the accuracy and reproducibility of outputs.

A typical radiomics workflow includes 4 main steps: image acquisition, tissue delineation, feature extraction and analysis. Clinical studies have shown that changes to these can reduce the number of robust features to 6 – 43% [14], [42]. Some steps depend on expertise (tissue delineation) or research question (analysis), but others can be standardised (image acquisition and feature extraction) [43], [44]. We have shown preclinical analysis to be more sensitive to these changes with 0.2–22% robust features identified.

Preclinical CBCT scans are acquired at lower energies than used clinically [15], [45], CBCT scan quality is known to have scattering and beam hardening artefacts in comparison to CT scans causing additional variabilities between scans [20]. Reduction of variabilities during image acquisition was achieved through use of a single, high imaging energy (60 kV). Advanced imaging methods such as dual-energy CT (DECT) improve image quality and could potentially reduce variabilities in radiomics analysis. However, imaging doses associated with preclinical DECT (60 cGy) are higher than single energy exposures (2.4 cGy) and repeated longitudinal imaging may have increased biological implications [46], [47].

Studies also recommend standardising image intensity discretisation through bin widths as a normalisation step for comparative analysis [48], [49], [50]. A fixed bin width was used for intensity discretisation for filtered features [51]. Our analysis identified bin widths of 25 or 50 to have the most robust features for analysis. Changing the slice thickness or pixel size can also reduce the impact of noise within the scans for the extraction of more reproducible and robust features [48]. First order, GLCM and GLRLM feature classes were the most robust to changes in slice thickness in agreement with other studies [48], [52]. However, altering the slice thickness during analysis caused changes to shape features which could significantly impact analysis. Further normalisation methods may be of interest for future preclinical radiomics studies [53], [54].

Studies have shown different segmentation volumes have a more significant effect on CT-derived features than MR- features [55]. [56], [57], [58] Roy *et al* showed that volume size had the largest influence on GLSZM features followed by GLCM, GLRLM and NGTDM features [28]. Some clinical analysis excludes tumours if they have a volume under a defined limit [56], [57], [58]. Segmentation volumes are typically smaller in preclinical models making them more challenging to delineate and contain fewer voxels or quantitative information for analysis. Our study is the first to evaluate the volume effect on preclinical radiomics outputs. Similar to clinical results, GLCM, GLSZM and NGTDM features were affected the most by changes in volume. As some features classes are more heavily influenced or dependent on volume to maximise reliability, first order and GLDM features should be used for analysis, or similar segmentation volumes should be compared [28], [56].

In clinical analysis, tumour volume has been shown to complement texture analysis of intra-tumoral heterogeneity [57]. Our results have determined 54 features highly correlated to changes in volume (Supplementary Table 2). Removing features dependent on volume changes should therefore be excluded from studies assessing tumour heterogeneity.

Phantoms are invaluable to radiation research to mimic tissue texture and density without repeated imaging dose to human or animal subjects [59]. Through the inclusion of a density phantom, similar to that of soft tissue (solid water) and bone (PVC), we demonstrated preclinical radiomics can differentiate between density changes. NGTDM features were further analysed as understandable texture properties [60], [61]. The creation of a dedicated preclinical radiomics phantom with differing densities and textural components may be more applicable for comparison of texture outputs with tissue equivalents.

Whilst our study provides a thorough analysis of robust and reliable features for preclinical radiomics, it has several limitations. Shape features was excluded from the repeatability and reproducibility analysis to remove user bias from manual contouring methods. Results from tumour models only provide proof of principle in extracting useful information from preclinical scans with additional analysis required to correlate features to biological parameters. This study is the first effort to optimise and standardise preclinical CBCT-radiomics analysis with further scope to compare radiomics outputs between research centres and across imaging modalities [62].

We present the first preclinical CBCT-radiomics workflow comparing changes to the repeatability and reproducibility of features across image acquisition, pre-processing parameters and segmentation sizes. Our results recommend that preclinical CBCT scans should be acquired at higher imaging energy (60 kV) and features extracted using a set bin width (25) and slice thickness (0.26 mm). Feasibility of extracting meaningful data was validated in a multi-texture phantom and preclinical models of NSCLC. Our data demonstrates that preclinical radiomics analysis is a novel tool that has the potential to develop imaging biomarkers to support the wider application of radiomics.

## Funding

KHB is supported by a Training Fellowship from the National Centre for the Replacement Reduction and Refinement of Animal in Research (NC3Rs, NC/V002295/1). NP is supported through a grant from the Northern Ireland Health and Social Care Trust R&D division (COM/4964/14). MG and KTB are supported by the Medical Research Council (MR/V009605/1).

## Declaration of Competing Interest

The authors declare that they have no known competing financial interests or personal relationships that could have appeared to influence the work reported in this paper.
