# Supplementary material for: Development and optimisation of a preclinical cone beam computed tomography-based radiomics workflow for radiation oncology research
Source: Phys Imaging Radiat Oncol. 2023 May 16;26:100446. doi: 10.1016/j.phro.2023.100446 (PMC10213103; doi:10.1016/j.phro.2023.100446)
Supplement: Supplementary data 1 [file mmc1.docx]

**Supplementary Figures**

**
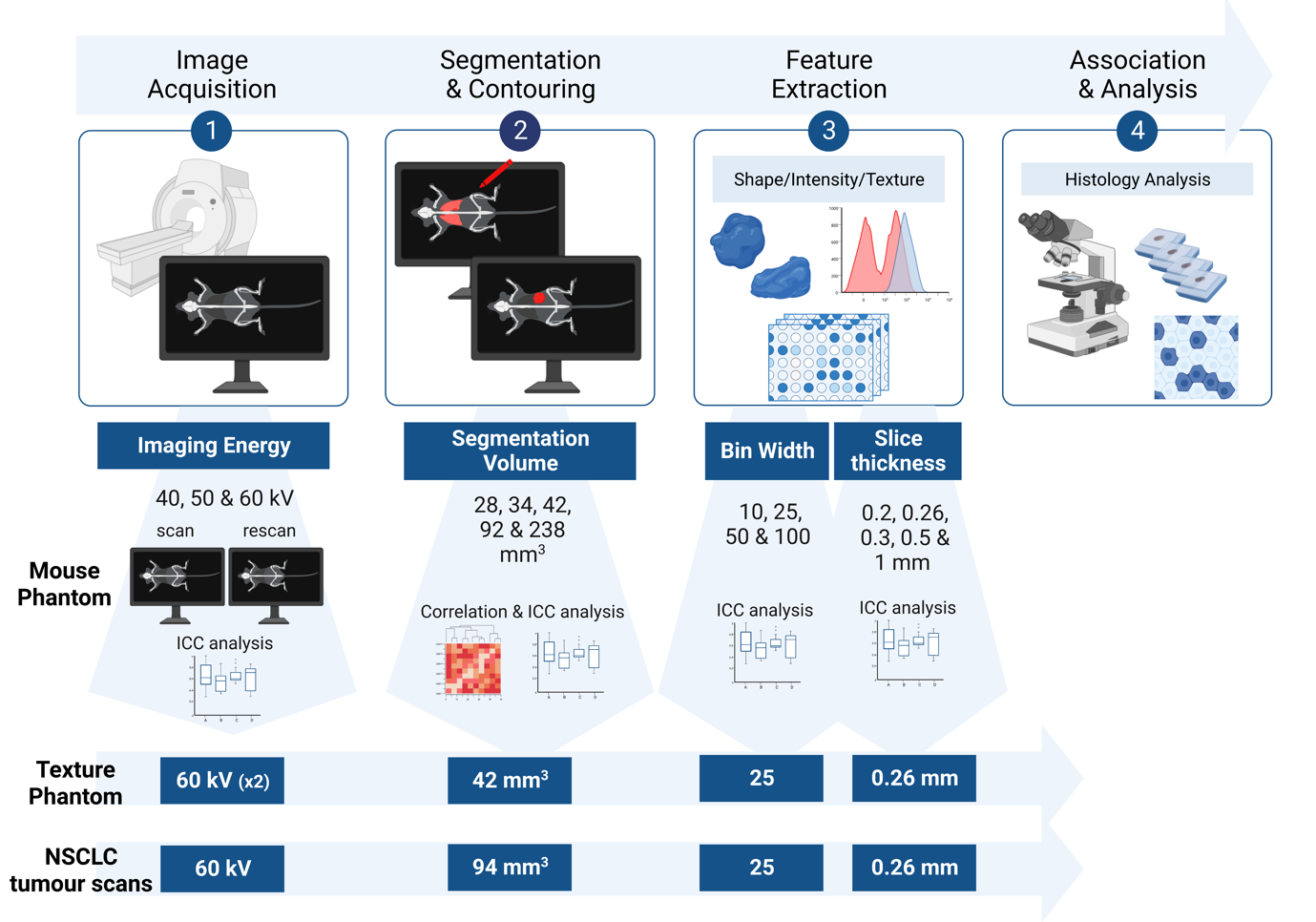
**

**Supplementary Figure 1: Overview of radiomics workflow and variables assessed within this study.** A 3D printed mouse phantom was used for workflow optimization through scan-rescan tests. A texture phantom was used to determine changes to textural features from preclinical CBCT radiomics analysis. Feasibility of application to preclinical datasets was completed using non-small cell lung cancer (NSCLC) tumour scans. Created with BioRender.com.

**Supplementary Figure 2: CBCT scans with attached contours for different segmentation volumes.**

Panel A: Axial view of the mouse phantom imaged at 60 kV on a CBCT scanner with the segmentation of 28, 34, 42, 92, and 238 mm^3^ shown in red.

Panel B: Corresponding coronal CBCT scans of the mouse phantom imaged at 60 kV with volumes shown in red.

**
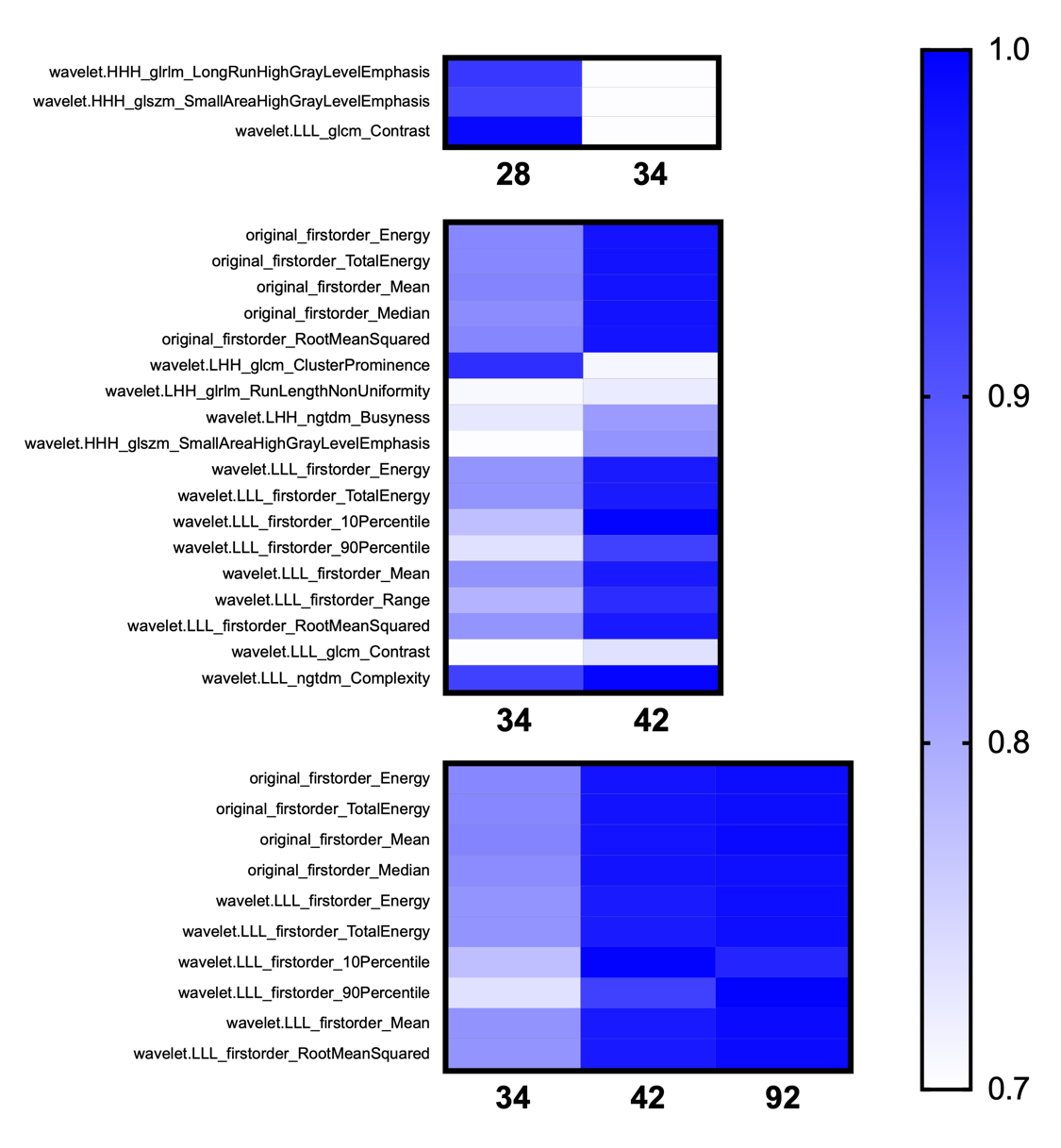
**

**Supplementary Figure 3: Heatmaps for overlapping features across different segmentation sizes.** Robust features with an ICC lower confidence interval >0.7 were compared and overlapping features presented in each heatmap.


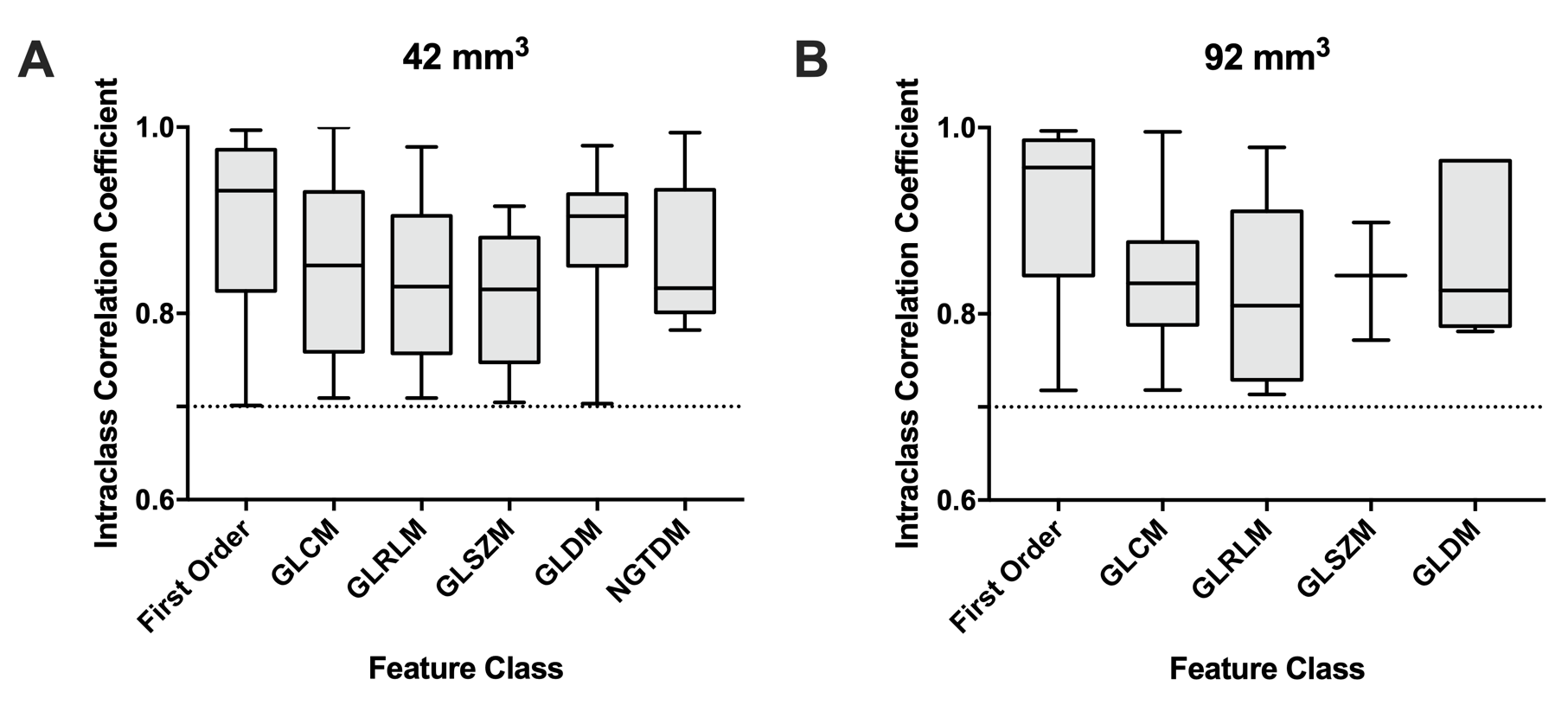


**Supplementary Figure 4: Further breakdown of robust radiomics feature for segmentation volumes of 42 mm^3^ and 92 mm^3^.**

Panel A: Boxplot to present the lower confidence intervals of ICC values for 42 mm^3^ volumes by radiomics feature class.

Panel B: Boxplot to present the lower confidence intervals of ICC values for 92 mm^3^ volumes by radiomics feature class.
