# Supplementary material for: Development and optimisation of a preclinical cone beam computed tomography-based radiomics workflow for radiation oncology research
Source: Phys Imaging Radiat Oncol. 2023 May 16;26:100446. doi: 10.1016/j.phro.2023.100446 (PMC10213103; doi:10.1016/j.phro.2023.100446)
Supplement: Supplementary data 2 [file mmc2.docx]

**Supplementary Data**

| **Manufacturer** | Xstrahl Life Sciences |
| --- | --- |
| **Platform** | SARRP |
| **Image Modality** | CBCT |
| **Device** | Tesla C2075 |
| **Detector Panel** | Perkin Elmer XRD 0822 AP3 |
| **Imaging Energy (kV)** | 40, 50, 60 |
| **Projections** | 0° - 359° |
| **Slice Thickness (mm)** | 0.26 |
| **Pixel Spacing (mm)** | 0.26/0.26 |
| **Reconstruction** | Backprojection without postfiltering |
| **Intensity Range** | 0 – 32770 |

**Supplementary Table 1: Details of the CBCT scanner used for analysis.**
