# Supplementary material for: Development and optimisation of a preclinical cone beam computed tomography-based radiomics workflow for radiation oncology research
Source: Phys Imaging Radiat Oncol. 2023 May 16;26:100446. doi: 10.1016/j.phro.2023.100446 (PMC10213103; doi:10.1016/j.phro.2023.100446)
Supplement: Supplementary data 3 [file mmc3.docx]

**Supplementary Data**

|  |  | **Robust features** | |
| --- | --- | --- | --- |
|  | **Correlation Coefficient** | **42 mm^3^** | **92 mm^3^** |
| original_shape_LeastAxisLength | 0.9868414 |  |  |
| original_shape_MajorAxisLength | 0.9867483 |  |  |
| original_shape_Maximum2DDiameterColumn | 0.9885362 |  |  |
| original_shape_Maximum2DDiameterRow | 0.9863885 |  |  |
| original_shape_Maximum2DDiameterSlice | 0.9863877 |  |  |
| original_shape_Maximum3DDiameter | 0.9873058 |  |  |
| original_shape_MinorAxisLength | 0.9869692 |  |  |
| original_shape_SurfaceArea | 0.9974264 |  |  |
| original_shape_VoxelVolume | 1.0000000 |  |  |
| original_firstorder_Energy | 0.9999969 | x | x |
| original_firstorder_TotalEnergy | 0.9999975 | x | x |
| original_firstorder_Entropy | 0.9934967 |  |  |
| original_firstorder_90Percentile | 0.9730258 | x | x |
| original_firstorder_Mean | 0.8719513 | x | x |
| original_firstorder_Median | 0.8669138 | x | x |
| original_firstorder_InterquartileRange | 0.9983591 | x |  |
| original_firstorder_MeanAbsoluteDeviation | 0.9896331 | x |  |
| original_firstorder_RobustMeanAbsoluteDeviation | 0.9938368 | x |  |
| original_firstorder_RootMeanSquared | 0.8769417 | x | x |
| original_firstorder_Skewness | 0.9230221 |  |  |
| original_firstorder_Variance | 0.9773407 | x |  |
| original_glcm_ClusterProminence | 0.9300029 |  |  |
| original_glcm_ClusterShade | 0.9025638 |  |  |
| original_glcm_ClusterTendency | 0.9844567 | x |  |
| original_glcm_Correlation | 0.9950250 | x | x |
| original_glcm_JointEntropy | 0.9332424 |  | x |
| original_glcm_Imc1 | 0.8864900 |  |  |
| original_glcm_Idm | 0.9867387 |  |  |
| original_glcm_Idmn | 0.8689880 |  |  |
| original_glcm_Id | 0.9801680 |  | x |
| original_glcm_Idn | 0.8157036 | x |  |
| original_glcm_InverseVariance | 0.9626121 |  | x |
| original_glcm_SumEntropy | 0.9936403 |  |  |
| original_glcm_SumSquares | 0.9781706 |  |  |
| original_glrlm_GrayLevelNonUniformity | 0.9994550 | x |  |
| original_glrlm_GrayLevelVariance | 0.9770646 | x |  |
| original_glrlm_LongRunEmphasis | 0.9763782 |  |  |
| original_glrlm_RunEntropy | 0.9922018 |  |  |
| original_glrlm_RunLengthNonUniformity | 0.9999992 |  |  |
| original_glrlm_RunVariance | 0.9790742 |  |  |
| original_glszm_GrayLevelNonUniformity | 0.9993261 |  |  |
| original_glszm_GrayLevelVariance | 0.9728876 |  |  |
| original_glszm_LargeAreaEmphasis | 0.9765296 |  |  |
| original_glszm_SizeZoneNonUniformity | 0.9999247 |  |  |
| original_glszm_ZoneEntropy | 0.9829776 |  |  |
| original_glszm_ZoneVariance | 0.9722127 |  |  |
| original_gldm_DependenceEntropy | 0.9800163 |  |  |
| original_gldm_DependenceNonUniformity | 0.9999347 |  |  |
| original_gldm_DependenceVariance | 0.9662047 |  |  |
| original_gldm_GrayLevelNonUniformity | 0.9994633 |  |  |
| original_gldm_GrayLevelVariance | 0.9774761 | x |  |
| original_gldm_LargeDependenceEmphasis | 0.9743254 |  |  |
| original_ngtdm_Busyness | 0.9851212 | x |  |
| original_ngtdm_Complexity | 0.9870953 |  |  |

**Supplementary Table 2: List of radiomics features highly correlated to increasing segmentation volume.** Features highlighted in blue display features which have been determined as reliable through scan-rescan analysis at 42 and 92 mm^3^.
