# Supplementary material for: Development and optimisation of a preclinical cone beam computed tomography-based radiomics workflow for radiation oncology research
Source: Phys Imaging Radiat Oncol. 2023 May 16;26:100446. doi: 10.1016/j.phro.2023.100446 (PMC10213103; doi:10.1016/j.phro.2023.100446)
Supplement: Supplementary data 4 [file mmc4.docx]

**Supplementary Data**

**Supplementary Table 3: Robust radiomics features from CBCT scans imaged at 60 kV with a segmentation volume of 42mm^3^.** 119 features were found to be robust and suitable for use in preclinical radiomics analysis. First order = 43, GLCM = 34, GLRLM = 15, GLSZM = 9, GLDM = 10, NGTDM = 8.
