# Supplementary material for: Development and optimisation of a preclinical cone beam computed tomography-based radiomics workflow for radiation oncology research
Source: Phys Imaging Radiat Oncol. 2023 May 16;26:100446. doi: 10.1016/j.phro.2023.100446 (PMC10213103; doi:10.1016/j.phro.2023.100446)
Supplement: Supplementary data 5 [file mmc5.docx]

**Supplementary Data**

**Supplementary Table 4: Robust radiomics features from CBCT scans imaged at 60 kV with a segmentation volume of 92mm^3^.** 57 features were found to be robust and suitable for use in preclinical radiomics analysis. First order = 25, GLCM = 16, GLRLM = 7, GLSZM = 3, GLDM = 6.
