# Supplementary material for: Development and optimisation of a preclinical cone beam computed tomography-based radiomics workflow for radiation oncology research
Source: Phys Imaging Radiat Oncol. 2023 May 16;26:100446. doi: 10.1016/j.phro.2023.100446 (PMC10213103; doi:10.1016/j.phro.2023.100446)
Supplement: Supplementary data 6 [file mmc6.docx]

**Supplementary Data**

| **Bin Width** | **Slice Thickness** | **Segmentation Size** |  |
| --- | --- | --- | --- |
|  |  |  |  |
| original_firstorder_Energy | original_firstorder_TotalEnergy | original_firstorder_10Percentile |  |
| original_firstorder_TotalEnergy | original_firstorder_10Percentile | original_firstorder_Mean |  |
| original_firstorder_10Percentile | original_firstorder_90Percentile | wavelet.LHL_firstorder_Entropy |  |
| original_firstorder_90Percentile | original_firstorder_Mean | wavelet.LHL_firstorder_RootMeanSquared |  |
| original_firstorder_Mean | original_firstorder_Median | wavelet.LHL_firstorder_Variance |  |
| original_firstorder_Median | original_firstorder_RootMeanSquared | wavelet.HHH_firstorder_Minimum |  |
| original_firstorder_InterquartileRange | original_firstorder_Kurtosis | wavelet.HHH_firstorder_Kurtosis |  |
| original_firstorder_Range | wavelet.LLL_firstorder_TotalEnergy | wavelet.LLL_firstorder_Minimum |  |
| original_firstorder_MeanAbsoluteDeviation | wavelet.LLL_firstorder_10Percentile | wavelet.LLL_firstorder_10Percentile |  |
| original_firstorder_RobustMeanAbsoluteDeviation | wavelet.LLL_firstorder_90Percentile | wavelet.LLL_firstorder_Mean |  |
| original_firstorder_RootMeanSquared | wavelet.LLL_firstorder_Mean | wavelet.LHL_glcm_Contrast |  |
| original_firstorder_Kurtosis | wavelet.LLL_firstorder_Median | wavelet.LHL_glcm_DifferenceAverage |  |
| original_firstorder_Variance | wavelet.LLL_firstorder_RootMeanSquared | wavelet.LHL_glcm_DifferenceEntropy |  |
| wavelet.LLH_firstorder_10Percentile | wavelet.LLL_glcm_Contrast | wavelet.LHL_glcm_DifferenceVariance |  |
| wavelet.LLH_firstorder_InterquartileRange | wavelet.LLL_glcm_Correlation | wavelet.LHL_glcm_SumSquares |  |
| wavelet.LLH_firstorder_MeanAbsoluteDeviation | wavelet.LLL_glcm_DifferenceVariance | wavelet.LHH_glcm_ClusterProminence |  |
| wavelet.LLH_firstorder_RobustMeanAbsoluteDeviation |  | wavelet.HHH_glcm_Autocorrelation |  |
| wavelet.LLH_firstorder_Variance |  | wavelet.HHH_glcm_JointAverage |  |
| wavelet.LHL_firstorder_Energy |  | wavelet.LLL_glcm_Contrast |  |
| wavelet.LHL_firstorder_TotalEnergy |  | wavelet.LLL_glcm_DifferenceVariance |  |
| wavelet.LHL_firstorder_RootMeanSquared |  | wavelet.LHL_glrlm_GrayLevelVariance |  |
| wavelet.LHL_firstorder_Variance |  | wavelet.LHL_glrlm_RunEntropy |  |
| wavelet.HLL_firstorder_InterquartileRange |  | wavelet.HHH_glrlm_HighGrayLevelRunEmphasis |  |
| wavelet.HLL_firstorder_Range |  | wavelet.HHH_glrlm_LongRunHighGrayLevelEmphasis |  |
| wavelet.HHL_firstorder_Maximum |  | wavelet.HHH_glrlm_ShortRunHighGrayLevelEmphasis |  |
| wavelet.HHH_firstorder_Minimum |  | wavelet.HHH_glszm_HighGrayLevelZoneEmphasis |  |
| wavelet.HHH_firstorder_Kurtosis |  | wavelet.HHH_glszm_SmallAreaHighGrayLevelEmphasis |  |
| wavelet.LLL_firstorder_Energy |  | wavelet.LHH_gldm_GrayLevelVariance |  |
| wavelet.LLL_firstorder_TotalEnergy |  | wavelet.HHH_gldm_HighGrayLevelEmphasis |  |
| wavelet.LLL_firstorder_Minimum |  | wavelet.HHH_gldm_SmallDependenceHighGrayLevelEmphasis |  |
| wavelet.LLL_firstorder_10Percentile |  | wavelet.LHH_ngtdm_Complexity |  |
| wavelet.LLL_firstorder_90Percentile |  |  |  |
| wavelet.LLL_firstorder_Mean |  |  |  |
| wavelet.LLL_firstorder_Median |  |  |  |
| wavelet.LLL_firstorder_Range |  |  |  |
| wavelet.LLL_firstorder_MeanAbsoluteDeviation |  |  |  |
| wavelet.LLL_firstorder_RobustMeanAbsoluteDeviation |  |  |  |
| wavelet.LLL_firstorder_RootMeanSquared |  |  |  |
| wavelet.LLL_firstorder_Variance |  |  |  |
| original_glcm_Correlation |  |  |  |
| original_glcm_Idn |  |  |  |
| wavelet.LLL_glcm_Correlation |  |  |  |
| wavelet.LLL_glcm_Idmn |  |  |  |
| wavelet.LLL_glcm_Idn |  |  |  |
| wavelet.LLL_ngtdm_Coarseness |  |  |  |
| **n = 45** | **n = 16** | **n = 31** |  |

**Supplementary Table 5: List of repeatable and reproducible features for different bin widths, slice thickness and segmentation sizes.**
